# What are cascading disasters?

**DOI:** 10.14324/111.444/ucloe.000003

**Published:** 2019-08-08

**Authors:** David Alexander, Gianluca Pescaroli

**Affiliations:** 1 University College London, London, UK

**Keywords:** built environment, cascading disasters, cascading effects, interdependencies, critical infrastructure, complex systems, scenarios, the environment, policy and law

## Abstract

Cascades have emerged as a new paradigm in disaster studies. The high level of dependency of modern populations on critical infrastructure and networks allows the impact of disasters to propagate through socio-economic systems. Where vulnerabilities overlap and interact, escalation points are created that can create secondary effects with greater impact than the primary event. This article explains how complexity can be categorised and analysed in order to find those weak points in society that enable cascading impacts to develop. Scenarios can be used to identify critical dependencies and guide measures designed to increase resilience. Experience suggests that many potential impacts of cascading disasters remain uninvestigated, which provides ample scope for escalation of impacts into complex forms of crisis.

Thirty-five years ago a group of scholars presented evidence that hazards are not the true cause of disasters but merely the trigger. Until that point, the ‘orthodox’ model had involved a simple linear relationship: hazards, such as earthquakes, floods or industrial explosions, acted upon vulnerability (of people, assets and activities) to create risk, which periodically turned into disaster. One of the most important concepts in this formulation was the magnitude-frequency rule, which, for natural hazards, stated that the larger the event, the less frequently it would occur. Kenneth Hewitt and his colleagues turned the orthodox model on its head. The root cause of disaster, they argued, is vulnerability [1].

Global change has convinced many scientists that the magnitude-frequency relationship for natural hazards involves trends and is not static [2]. Vulnerability is dynamic as well. Whereas the ‘hazardscape’ or ‘hazardousness of place’, the geographical manifestation of hazards, is complicated by shifting mean frequencies and the action of one hazard upon another, the trends and interactions of vulnerability are vastly more complex. Moreover, they are difficult to study, for vulnerability, like friction, is a property that fully manifests itself only when it is mobilised by the application of a force. By the time one can characterise it, it has been transformed into impact. Hence, most studies of vulnerability are hypothetical and predictive, just as they are for risk, which is not surprising, as vulnerability is the dominant component of risk [3].

Studies of the interaction between hazards got underway in the 1960s and 1970s. It became apparent, for example, that a single earthquake could produce as many as 20,000 landslides [4]. Hurricanes could spawn multiple tornadoes; rock avalanches could dam rivers and cause catastrophic outburst floods. Studies of the interaction between forms of vulnerability have been less common and have been relatively late to appear, yet in the last 70 years world population has tripled, putting more people, their assets and their activities at risk. In the period 1970–3 the wealth differential began to increase, which it has continued to do ever since, leading to gross disparities in income and opportunity. Climate change may ramp up hazards, but a potentially greater problem is our increasing dependency upon networks, and the consequences if they fail us.

Consider the example of space weather. In 2012 a coronal mass ejection of the magnitude of that which occurred in 1859 narrowly missed the Earth [5]. Had it reached us, we might have endured widespread disruption of global positioning systems, satellites, telecommunications and electricity supply. A ‘Carrington event’ of this magnitude would arrive with 150 billion times the energy of the Hiroshima nuclear bomb [6]. Geomagnetic-induced currents and ionising radiation would potentially wreak havoc with technology. Unfortunately, the extent of dependency on the affected technologies is still poorly mapped. Hence, there would be some unpleasant surprises.

A ‘Carrington event’, named after the amateur astronomer who discovered solar flares, is an example of something that could trigger a cascading disaster. The simplest analogy for a cascading disaster is a line of toppling dominoes, in which an impact is propagated through a series of different domains [7]. However, there is much more to the phenomenon than this. Vulnerability to disaster manifests itself in a series of categories: physical and structural, environmental, social, psychological, institutional, and so on. They do not develop independently of one another but interact on many different time and space scales. The nested set of adaptation cycles that evolve in response to the spread of vulnerabilities is known as ‘panarchy’, but the adaptation is unlikely to be complete and destructive interactions between systems of vulnerability can lead to ‘panarchical collapse’, and hence disaster. Where vulnerabilities coalesce and overlap, escalation points are formed (Fig. 1). In cascading disasters it is common for the secondary effects to be new sources of impact, which may be more devastating than the original trigger [8].

The M9 earthquake that struck northeast Japan on 11 March 2011 killed about 100 people. However, the resulting tsunami killed at least 19,360 people (and a further 2569 were listed as missing). The nuclear radiation release from the Fukushima Dai’ichi power plant may in the end be the enduring legacy of the disaster, and the clear-up involves problems that will take up to half a century to resolve. The Tōhoku triple disaster was one of the best examples of a cascading event. It created a vast field of debris in the middle of the Pacific Ocean, it stopped automotive production in European plants, and it led to radioactive contamination and social problems that continue without end.

**Figure 1:**
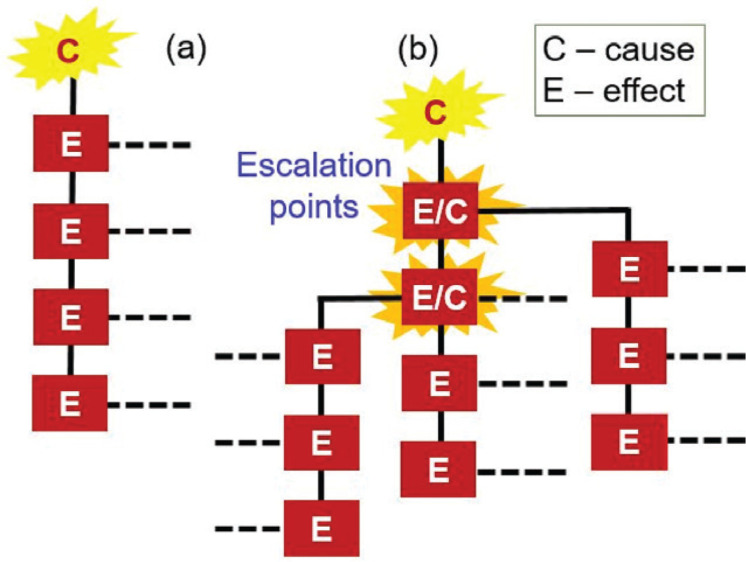
Cascading causes (C) and effects (E). (a) Linear path of events in disasters, and (b) non-linear escalation of cascading disasters where E/Cs represent subsidiary disasters [7].

From now onwards, most disasters will be cascading events to a greater or lesser extent. This is because of the high degree of dependency on networks of modern society. Hazards of all kinds, from storms to cyberattacks, threaten the critical infrastructure that we depend upon to conduct our daily activities and live in comfort and security. Failures can propagate through critical infrastructure and between its various categories [9]. Among these, one can single out the generation and distribution of electricity. Modern ‘black-sky thinking’ tells us that wide-area, prolonged power failure will have knock-on effects in all the other categories, from food storage to banking, water and sewerage to fuel supply, telecommunications to transportation [10]. Beyond these are the largely uncharted waters of the human social and economic consequences of the failures.

Even in the domain of technology, it is salutary to reflect that virtually all means of mass communication depend on electricity to make them work. Studies of diesel generator capacity suggest that it is both insufficient and a highly unreliable substitute for power from the grid [11]. Despite vigorous assurances from the electricity industry that ‘everything is under control’, wide-area power failures do occur, at a rate of about one a year worldwide, and they usually take days or weeks to rectify. No electricity grid is entirely immune to all the threats that it faces: cyberattacks and coordinated-sabotage terrorism, major storms and flooding, space weather, even sudden excessive demand, for example during a very hot summer. Rather than taking electricity for granted, we should be developing scenarios of how we would cope without it for extended periods of time.

Recently, a magnitude scale for cascading disasters has been proposed (Fig. 2) [12]. The purpose of this is to provide some comparability so that events can be cross-referenced in order to adapt lessons and experience from one case to another, and thus build better planning scenarios. The Cascading Disasters Research Group at University College London has been working on strategies for the identification of the vulnerability paths by which cascades propagate. This involves using gap analyses to understand the areas in which planning and measures are lacking or ineffective. The group has also identified five kinds of complex disaster impact, as follows (Fig. 3). (a) Compound risks involve the interaction of different extreme events or their drivers, such as storms, climate change and sea-level rise. They can also involve events that are merely coincident in time, such as an earthquake during a period of intense cold weather. (b) Interacting risks involve environmental drivers that can give primary and secondary impacts, as with seismically induced mass movements. (c) Interconnected risks cover the interaction of natural and human systems. This category includes the so-called ‘na-tech’ events, in which a natural impact triggers a technological one. For example, in the Czech Republic in 2002 and during Hurricane Harvey in Texas in 2017, floods inundated industrial premises and caused fires, explosions and toxic smoke emissions. (d) Cascading impacts disrupt critical infrastructure and closely linked organisational systems. (e) Finally, complex disasters may involve elements of any or all of the previous four categories [13].

**Figure 2:**
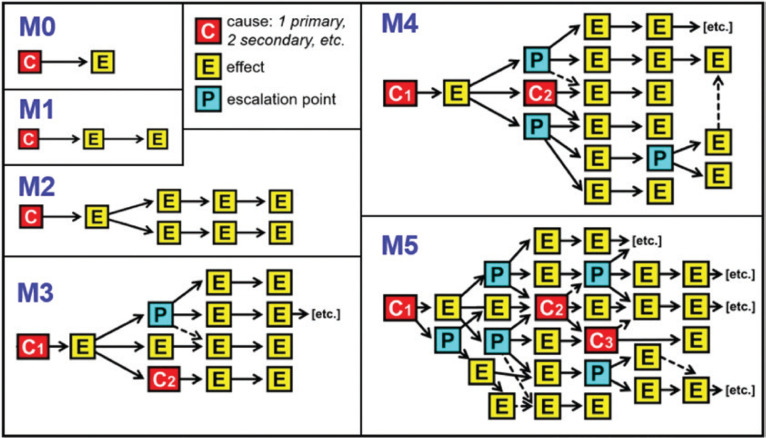
Magnitude scale for cascading disasters.

**Figure 3:**
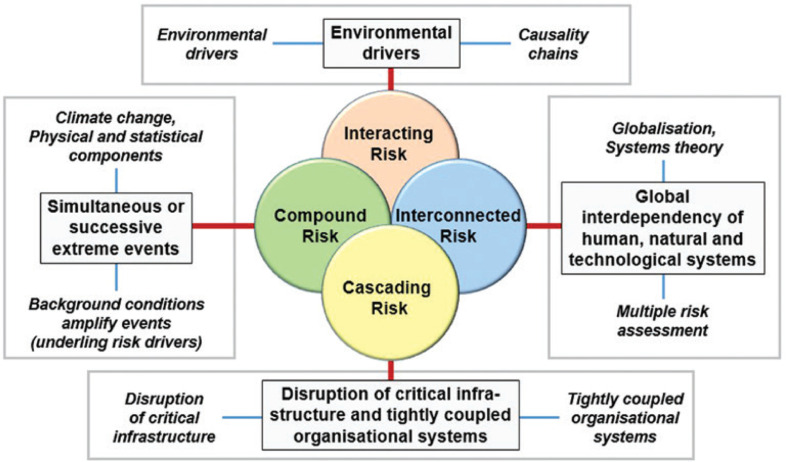
Compound, interconnected, interacting and cascading disasters.

The remedy for cascading disasters begins by recognising that they are the new reality and must be coped with. Fault and event trees can be compiled in order to investigate the vulnerability paths by which cascading impacts are propagated. For example, in the United Kingdom, hospitals are required to have generators and 11 days’ diesel fuel supply to run them. However, few generators are adequately tested, many are poorly maintained, and few are capable of running at full speed for prolonged periods of time [14]. This raises the questions of what will be the consequences if normal and back-up electricity supplies fail, and what can be done both to reduce the risk and to cope with potential consequences.

Dependencies on networks need to be identified and quantified. When, in April 2010, ash from the Icelandic volcano Eyjafjallajökull led to the grounding of civil aviation at 70% of Europe’s airports for almost a week, attention was focussed on the meteorology, volcanology, remote sensing and air traffic control aspects. Many other effects of the crisis went largely unobserved. Eight and a half million passengers were stranded. There was massive imbalance in the supply of and demand for ground and sea transportation and hotel accommodation. Critical supplies (such as bone marrow for transplants) could not be air freighted. Industries that are dependent on air transportation suffered major losses, and these included horticultural and agricultural enterprises, not merely airlines and airport service companies. Cultural and business activities were affected. Given that in the mid-1820s Eyjafjallajökull erupted for 13 months, and that it is one of the less powerful Icelandic volcanoes, there is an urgent need for planning scenarios that evaluate the effects of having to do without air travel for months on end [15].

New cross-disciplinary literature has been developed to support the understanding of cascading crises in the global interconnected system, including cross-domain modelling of interdependent systems, decision support systems and impact assessments [16]. These academic works tend to merge together quantitative and qualitative methodologies, producing both algorithms and scenarios for end users. The predictive evaluation of cascading effects has been improved and applied to multi-hazard analysis, including new modelling tools for assessing disruptions and losses, for example [17, 18]. Moreover, new considerations and methodologies have been elaborated to understand better interdependencies and complexity, such as integrations between linear and networked risk assessments [19], dynamic measures of criticality [20], and resilience of urban environments in climate change scenarios [21, 22]. 

The pace of change in the modern world is now faster than it has ever been in human history. On a planet that is more crowded with people than ever before, we face some formidable challenges on how to provide safety and security. Complex systems need to be understood in terms of their patterns of vulnerabilities and dependencies. The problem cannot be solved by technology alone. Indeed, technology can be part of the problem as much as it is a source of the solution. In a book on automation, David Noble observed that ‘...close inspection of technological development reveals that technology leads a double life, one which conforms to the intentions of designers and interests of power and another which contradicts them—proceeding behind the backs of their architects to yield unintended consequences and unanticipated possibilities’ [23]. With a robust theoretical framework, we can investigate the consequences and come to understand complexity and how to ensure that the possibilities are positive ones associated with protection, adaptation and foresight.

One final word about cascading crises needs to be added. In modern disasters, context is all important. Events, developments, policies and phenomena that are seemingly unconnected with the disaster may be fundamental to its outcome. For example, in modern neoliberal states there is a tendency to use fiscal austerity as a means of dismantling the welfare state. There have been notable increases in general vulnerability and the number of people who are destitute or desperately poor. In the world’s lowest-income countries, there has been some progress in lifting people out of absolute poverty, but success has been limited. The vulnerable include people with disabilities, the sick, the elderly and people with inadequate incomes. These are the people who are most likely to suffer the most serious effects of disaster, either directly or indirectly through the destruction or weakening of normal support mechanisms. Thus, disaster tends to ‘pick off’ the least able in society. This should be a powerful moral argument for revitalising the concept and practice of welfare, and making it proof against complex cascading impacts.

## Data Availability

No further data were used in addition to referenced works.
